# Sociodemographic, clinical and personal characteristics of patients with borderline personality disorder in a public general hospital in Lima, Peru during the first wave of the COVID-19 pandemic

**DOI:** 10.1192/bjo.2021.198

**Published:** 2021-06-18

**Authors:** Glauco Valdivieso

**Affiliations:** Instituto Peruano para el Estudio y Abordaje Integral de la Personalidad, Servicio de Psiquiatría, Hospital de Emergencias Villa EL Salvador

## Abstract

**Aims:**

To describe the main characteristics of adolescent and adult patients with Borderline Personality Disorder (BPD) treated in Emergency and Hospitalization services of Villa El Salvador Emergency Hospital during the first wave of the COVID-19 pandemic in Lima, Peru.

**Method:**

An analysis of 17 cases of patients with BPD according to DSM 5 criteria was carried out in SISGALEN PLUS software database that have been evaluated in the Emergency and Hospitalization areas during the first wave of the COVID-19 pandemic. Sociodemographic, clinical and personal variables were taken into account. A descriptive analysis of frequencies and proportions was carried out in SPSS 24.0 software.

**Result:**

Regarding sociodemographic variables, the average age was 27.47 (SD = 11.242), 82.4% single, 88.2% female, 52.9% from Villa El Salvador, 82.4% catholics, 76.5% have completed secondary school and 47.1% were housewives. For clinical variables, 64.7% located in the Emergency Service, 58.8% had no current diagnosis of COVID-19, 64.7% without medical comorbidity, 35.3% without psychiatric comorbidity, 52.9% with suicide attempt as the main reason for consultation, 52.9% without regular use of medications, 88.2% with psychopharmacological treatment; 70.6% received a psychiatric interview intervention; Regarding symptoms, all presented interpersonal problems, impulsivity, emotional instability and inappropriate anger, while 58.8% had alteration of identity and 94.1% had suicidality. For personal variables, 82.4% had no family history, 88.2% had no history of abuse or trauma, 52.9% had a history of substance use, and 88.2% had no previous hospitalizations.

**Conclusion:**

The most of patients with BPD were young adults, women, single, from Villa El Salvador, catholics, completed secondary school, housewives, from Emergency, no diagnosis of COVID-19, without medical or psychiatric comorbidity, consulted for suicide attempt, without habitual use of medications, with indicated psychopharmacological treatment, a psychiatric interview was conducted, they had active symptoms, history of substance use and no family history, abuse or hospitalizations.

